# The Fault in Our Astrocytes - cause or casualties of proteinopathies of ALS/FTD and other neurodegenerative diseases?

**DOI:** 10.3389/fmmed.2023.1075805

**Published:** 2023-02-16

**Authors:** Lynette M. Bustos, Rita Sattler

**Affiliations:** ^1^ School of Life Sciences, Arizona State University, Tempe, AZ, United States; ^2^ Barrow Neurological Institute, Phoenix, AZ, United States

**Keywords:** astrocytes, neurodegenaration, proteinopathies, ALS, FTD, Alzheimer's disease, Huntingtons’s disease, Parkinson’s disease

## Abstract

Many neurodegenerative diseases fall under the class of diseases known as proteinopathies, whereby the structure and localization of specific proteins become abnormal. These aberrant proteins often aggregate within cells which disrupts vital homeostatic and physiological cellular functions, ultimately contributing to cell death. Although neurodegenerative disease research is typically neurocentric, there is evidence supporting the role of non-neuronal cells in the pathogenesis of these diseases. Specifically, the role of astrocytes in neurodegenerative diseases has been an ever-growing area of research. Astrocytes are one of the most abundant cell types in the central nervous system (CNS) and provide an array of essential homeostatic functions that are disrupted in neurodegenerative diseases. Astrocytes can exhibit a reactive phenotype that is characterized by molecular changes, as well as changes in morphology and function. In neurodegenerative diseases, there is potential for reactive astrocytes to assume a loss-of-function phenotype in homeostatic operations such as synapse maintenance, neuronal metabolic support, and facilitating cell-cell communication between glia and neurons. They are also able to concurrently exhibit gain-of-function phenotypes that can be destructive to neural networks and the astrocytes themselves. Additionally, astrocytes have been shown to internalize disease related proteins and reflect similar or exacerbated pathology that has been observed in neurons. Here, we review several major neurodegenerative disease-specific proteinopathies and what is known about their presence in astrocytes and the potential consequences regarding cell and non-cell autonomous neurodegeneration.

## 1 Introduction

The concept of astrocytes was first introduced in the 1800’s by Rudolf Virchow, where he described “neuroglia” as connective material between neurons (Kettenmann and Verkhratsky, 2008). In the same century, Michael von Lenhossek branded these cells as “astrocyten” or “star cells” describing their star-like morphology. Astrocytes are one of the most abundant cell types in the central nervous system (CNS) and undergo a morphological and functional maturation process based on environmental cues from the surrounding microenvironment (Molofsky and Deneen, 2015; Khakh and Deneen, 2019). This maturation coincides with an increase in formation, density, and elimination of synapses that aids in neuronal circuitry maturation (Molofsky and Deneen, 2015). This intricate relationship between astrocytes and neurons was first proposed by Carl Ludwig Schleich in 1899 (Kettenmann and Verkhratsky, 2008). In addition to aiding in the formation and maintenance of synapses, astrocytes contribute to the blood-brain barrier, provide trophic support to neurons, and facilitate neuron-glia and glia-glia communication (Khakh and Deneen, 2019).

In the human cortex, astrocytes have been characterized by their distinct morphological properties. The four subtypes are interlaminar (located in layers I and II), protoplasmic (located in layers III and IV), varicose projections (located in layers V and VI), and fibrous (located in the white matter) (Oberheim et al., 2009; Vasile et al., 2017). Although studies use morphology as one defining characteristic of an astrocyte’s function within the brain, they have been recently categorized molecularly in the murine brain using single-cell transcriptomics; thus, defining specialized astrocyte subtypes between brain regions (Batiuk et al., 2020). Cortical astrocytes express, at higher levels, genes that encode proteins required for metabolism, gliotransmission and neurotransmitter uptake and recycling (Oberheim et al., 2009; Zhang et al., 2016; Vasile et al., 2017; Peteri et al., 2019).

In the same way astrocyte function is heterogeneous, so too is their activation and reactivity to physiological conditions and disease pathologies, respectively. Much work has demonstrated that astrocytes can take on neurotoxic and neuroprotective phenotypes in response to a CNS insult (Faulkner et al., 2004; Okada et al., 2006; Herrmann et al., 2008; Zamanian et al., 2012; Liddelow et al., 2017; Clarke et al., 2018). However, as technologies used in the field of astrocyte biology have advanced, the dualistic view of “A1 vs. A2” reactive astrocytes has blurred. The term “reactive astrogliosis” has recently been redefined as a process induced in a pathological context-specific manner that results in a loss or upregulation of homeostatic functions and/or the gain of new function(s) (Escartin et al., 2021). Therefore, the term “astrocyte reactivity” is the ability of astrocytes to adopt a discrete state in response to a specific disease-related pathology, and the term “astrocyte activation” is to be associated with a change to physiological conditions not due to a pathological source (Escartin et al., 2021). The consensus of the field is that astrocyte reactivity is most likely a secondary event triggered by an external signal at a specific timepoint in the pathological progression, within some contexts where the phenotypes are reversible (Escartin et al., 2021).

There is mounting evidence for the important role of astrocytes in disease pathogenesis of various neurodegenerative diseases. There is a critical loss of supportive astrocytic functions and in some contexts a toxic gain-of-function that causes astrocytes to not only affect the health of vulnerable neurons, but to develop a disease specific reactive phenotype. Recently, a study assessed astrocyte-derived toxic factors in an induced reactive state that has been previously shown to be detrimental to the health and survival of neurons and other glia (Guttenplan et al., 2021). The authors screened reactive and quiescent astrocytes and conditioned media (ACM) using mass spectrometry to assess for changes in protein abundance following activation *via* addition of pro-inflammatory cytokines, IL-1α, TNFα and C1q. There was an increase in C3 and other reactivity factors in both cells and ACM, but none facilitated cell death in their *in vitro* systems. In reactive ACM, other than known reactivity markers, lipoparticle proteins such as APOE and APOJ were upregulated. By immunodepleting reactive ACM of APOE and APOJ, the author’s observed a decrease in toxicity of reactive ACM. Furthermore, they described an upregulation of long-chain saturated free fatty acids in reactive ACM. Through conditional knockout of the metabolic enzyme ELOVL3, they were able to reduce long-chain saturated free fatty acid production and reduce reactive ACM toxicity towards neurons and oligodendrocytes in both *in vivo* and *in vitro* conditions, respectively. Demonstrating that reactive astrocytes, through the secretion of toxic lipids, contributes to neuronal death. However, clear direct evidence of this altered lipid metabolism occurring in neurodegenerative diseases is missing.

Astrocytes have a role in mediating or exacerbating pathological conditions, especially in the context of neurodegenerative diseases. In many neurodegenerative diseases, astrocytes have also shown evidence of pathological inclusions. It is not certain whether this is initiated by the disease itself directly affecting astrocytes and causing protein inclusions, or if it is a compensatory mechanism where astrocytes are internalizing pathological proteins released by or crowding around neurons. In this review, we will summarize what is currently known about how the distribution of these aberrant proteins causes disease-specific alterations in astrocytes, and how this might contribute to neuronal cell death (see Table 1). We will discuss the presence of astrocytic proteinopathies in the spectrum disease of Amyotrophic lateral sclerosis and Frontotemporal Dementia (ALS/FTD), as well as Alzheimer’s disease, Huntington’s disease and Parkinson’s disease.

**TABLE 1:** Neurodegenerative disease pathologies affecting astrocytes.

Disease	Proteinopathy	Human astrocytic inclusions?	Affected astrocyte functions in human tissue and model systems	References
ALS/FTD	TDP-43	Observed in the cytoplasm and radiating processes (identified as glial inclusions; unclear if astrocyte specific)	• Increase in GFAP expression	Nishihira et al. (2008), Cooper-Knock et al. (2012), Peng et al. (2020), Velebit et al. (2020)
			• Transcriptional reactive phenotype	
			• Impaired lipid metabolism, ß-adrenergic mediated aerobic glycolysis, and lactate production	
	FUS	Cytoplasmic and nuclear inclusions (identified as glial inclusions; unclear if astrocyte specific)	• Increase in GFAP expression and secreted factors (TNFα)	Hewitt et al. (2010), Suzuki et al. (2012), Kia et al. (2018)
	SOD1	Nuclear accumulations in the ventral horn of the spinal cord	• Increase in GFAP expression	Nagai et al. (2007), Yamanaka et al. (2008), Forsberg et al. (2011), Guttenplan et al. (2020)
			• Reactive transformation	
			• Secretion of motor neuron specific toxic factor	
			• Impaired glutamate uptake	
	*C9orf72* DPRs	Detected in iPSC-derived astrocytes	• Increase in GFAP expression	Shi et al. (2018), Birger et al. (2019), Varcianna et al. (2019), Guttenplan et al. (2020), Zhao et al. (2020), Taha et al. (2022)
			• Reactive transformation	
			• Impaired glutamate uptake	
			• Impaired EV secretion	
			• Oxidative stress	
	TAU	Observed in tufted astrocytes and astrocyte plaques	• Increase in GFAP expression	Bang et al. (2015), Hallmann et al. (2017)
			• Astrogliosis - reactive transformation	
			• Morphological changes (hypertrophy)	
Alzheimer’s disease	Amyloid-ß	Astrocytes found surrounded around Aß plaques	• Aß_42_ oligomers caused endosomal/lysosomal defects	Pike et al. (1995), Bates et al. (2002), Söllvander et al. (2016)
			• Increase in GFAP expression	
	Neurofibrillary Tangles - Tau	Observed in hilar astrocytes in the dentate gyrus	• Impaired mitochondrial motility and function	Yoshiyama et al. (2003), Forman et al. (2005), Richetin et al. (2020)
			• Impaired trafficking network	
	TDP-43	Not found, glial cytoplasmic inclusions are found in transferrin-positive oligodendrocytes	Not well described	Higashi et al. (2007)
Huntington’s disease	HTT Expansion Variant	HTT expansion variant fibrils found in the nucleus and cytoplasm of striatal astrocytes	• Reactive transformation	DiFiglia et al. (1997), Cooper et al. (1998), Shin et al. (2005), Faideau et al. (2010), Tong et al. (2014), Jansen et al. (2017), Diaz-Castro et al. (2019)
			• Impaired glutamate uptake and K^+^ buffering	
			• Morphological changes and hypertrophy	
			• Electrophysiological defects, (depolarized membrane potentials and lower membrane conductance)	
			• Altered functions in Ca^2+^ signaling, GPCR, and neurotransmitter regulation	
Parkinson’s disease	α-synuclein	Observed within the substantia nigra, midbrain, amygdala, thalamus, striatum and cerebral cortex	• Reactive transformation with pro-inflammatory response	Wakabayashi et al. (2000), Braak et al. (2007), Lee et al. (2010), Rostami et al. (2017), Russ et al. (2021)
			• Antigen-presenting phenotype (dependent on α-synuclein species)	
			• Impaired phagosomal-lysosomal machinery	
			• Impaired glutamate uptake	

## 2 Amyotrophic lateral sclerosis and frontotemporal dementia-associated proteinopathy

### 2.1 Amyotrophic lateral sclerosis-associated proteinopathy

Amyotrophic lateral sclerosis (ALS) is a fatal progressive neurodegenerative disorder that causes the loss of upper and lower motor neurons, leading to muscle atrophy and loss of muscle control, and ultimately the inability to walk and breath (Hardiman et al., 2017; Balendra and Isaacs, 2018). The onset of disease can occur between 40 and 70 years of age. The majority of ALS cases are categorized as sporadic (sALS), and just over 10% of sALS cases are associated with a genetic variant (Yun and Ha, 2020). Only a small subset, about 10% of ALS cases, are classified as familial (fALS), where about 70% of fALS cases are associated with a genetic variant (Yun and Ha, 2020). Over 50 potential genes have been associated with ALS, and the two most common genetic abnormalities are those found in the *C9orf72* and *SOD1* genes (Mejzini et al., 2019). The GGGGCC (G_4_C_2_) hexanucleotide repeat expansion (HRE) in the first intron of the gene *C9orf72,* is the most common genetic abnormality associated with both ALS and frontal temporal dementia (FTD) (DeJesus-Hernandez et al., 2011; Renton et al., 2011). ALS and FTD patient brains can exhibit multiple proteinopathies, dependent on the specific patient subgroup. Here, we will discuss ALS-associated proteinopathy, and then further discuss FTD-tau associated proteinopathy and several overlapping ALS/FTD proteinopathies.

Non-cell autonomous disease mechanisms in ALS have been well-accepted for over 2 decades, suggesting a significant contribution of the observed neurodegeneration is exacerbated by non-neuronal cells (Rothstein et al., 1996; Clement et al., 2003). A common pathogenic feature of ALS astrocytes is the loss-of-function mechanism that leads to neuronal glutamate excitotoxicity. In ALS patients, there is significant evidence demonstrating a decrease in glutamate transport within the cortex and spinal cord (Rothstein et al., 1992). The reduced expression of astrocytic glutamate transporter, EAAT2, is responsible for this phenomenon (Rothstein et al., 1996). Consequently, ALS astrocytes have a reduced ability to uptake glutamate at the synapse, leading already vulnerable motor neurons to also undergo glutamate excitotoxicity. More recently, a study proposed the astrocytic release of inorganic polyphosphate (polyP) as a motor neuron specific vulnerability in ALS/FTD (Arredondo et al., 2022). In this study, they utilized *in vitro* and *in vivo* models with *SOD1, TARDBP* and *C9orf72* ALS/FTD-related genetic variants to describe an excessive and ultimately toxic release of inorganic polyP by astrocytes. They further observed enrichment of polyP in astrocyte-like cells in postmortem spinal cord tissue from SOD1, C9orf72 and sALS patients. Ultimately, astrocytes have been shown to elicit both a cell autonomous and non-cell autonomous disease pathogenesis in both sporadic and familial forms of ALS leading to the death of motor neurons.

#### 2.1.1 SOD1 nuclear accumulations

**FIGURE 1:**
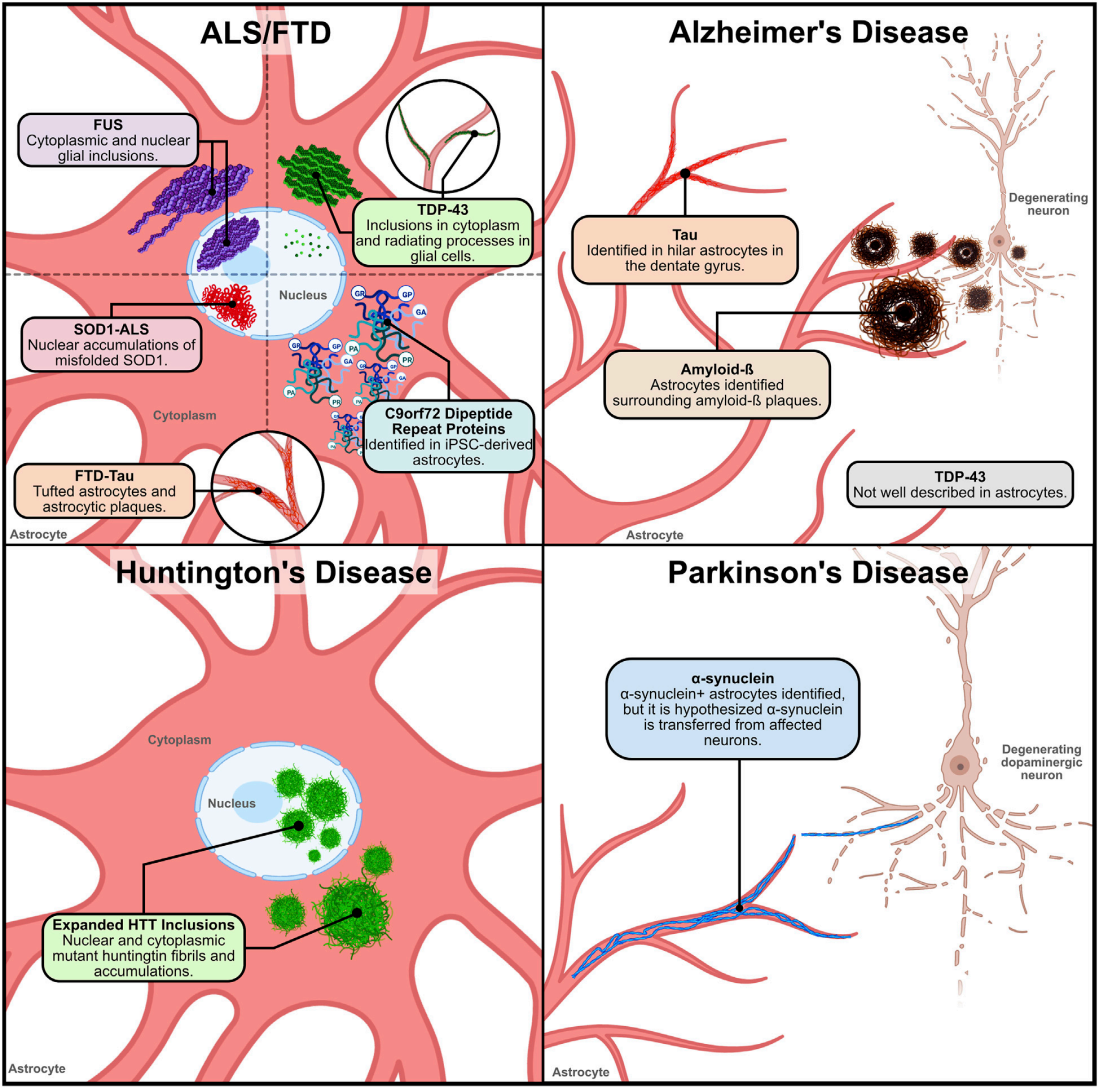
Location and distribution of neurodegenerative disease proteinopathies within and around astrocytes. 1) ALS/FTD – Various proteinaceous inclusions occur in ALS and FTD astrocytes. In ALS and FTD, TDP-43 inclusions have been found in the cytoplasm and radiating processes of glial cells. In ALS and FTD cases with FUS variants, cytoplasmic and nuclear glial inclusions have been identified. However, the identification of TDP-43 and FUS inclusions specifically in astrocytes has not been clarified. Nuclear accumulations of misfolded SOD1 caused by ALS-linked genetic variants have been identified in ventral horn astrocytes. In FTD-TAU, tufted astrocytes and astrocytic plaques have been observed in patient tissue. This pathology is similar to what has also been observed in AD tauopathy. and in C9orf72-ALS iPSC-derived spinal cord-like astrocytes the presence of DPRs were detected. 2) Alzheimer’s disease–In Alzheimer’s disease, astrocytes have been described to surround and internalize extracellular amyloid-ß (Aß) plaques. Reactive astrocytes found in postmortem AD tissue have been observed to have a significant Aß load. Tau is not expressed in astrocytes explicitly, so the source of tau in astrocytic accumulations in AD is unknown. However, tau accumulation in hilar astrocytes has been described. AD TDP-43 pathology has not been well characterized in astrocytes. 3) Huntington’s disease – In Huntington’s disease, expanded huntingtin (HTT) fibrils in the nucleus and cytoplasm of cortical and striatal astrocytes have been observed. Expanded HTT inclusions in astrocytes occurs early in the disease pathogenesis, suggesting that astrocytic disruptions may contribute to disease progression. 4) Parkinson’s disease – In Parkinson’s disease, α-synuclein^+^ inclusions in astrocytes of PD patient tissue found within the SN, midbrain, amygdala, thalamus, striatum and cerebral cortex. In early stages of disease pathogenesis, α-synuclein^+^ astrocytes are found in the amygdala, striatum, and thalamic nuclei projecting to the cortex. In the cortex, astrocytic α-synuclein^+^ inclusions are found in layers V and VI, with limited prevalence in layers III and IV. However, studies hypothesize that the α-synuclein found in astrocytes is possibly transferred from affected dopaminergic neurons. Figure created with Biorender.com.

Early works have primarily studied spinal cord astrocytes in the context of the first genetic abnormality associated with ALS—missense point mutations in the Superoxide Dismutase 1 (*SOD1*) gene. *SOD1*-ALS accounts for ∼12% of familial and ∼1% of sporadic cases (Renton et al., 2014). Although this genetic variation of ALS presents with progressive motor neuron degeneration, there is no TDP-43 pathology present. In *SOD1*-ALS, SOD1 variants undergo misfolding that causes structural and functional defects in catalytic activity and metal binding leading to a toxic gain-of-function (Huai and Zhang, 2019). Misfolded SOD1 variants have been observed to localize not only in motor neurons of the spinal cord, but also in the nuclei of astrocytes (see Figure 1), microglia and oligodendrocytes in the ventral horn (Forsberg et al., 2011). Early studies demonstrated that when SOD1 variants are expressed only in motor neurons of a chimeric mouse model, this is not sufficient to initiate neurodegeneration in the spinal cord (Clement et al., 2003). However, primary mouse astrocytes expressing SOD1^G93A^ variant reduced the survival of both wild type and SOD1^G93A^ motor neurons in co-culture conditions, eliciting a direct effect of neuronal degeneration (Di Giorgio et al., 2007). Additionally, primary mouse motor neuron cultures treated with preconditioned media from primary astrocytes from SOD1^G93A^ mice displayed a selective toxic effect on neuronal morphometry and survival (Nagai et al., 2007). When conditioned media from SOD1^G93A^ cortical neurons, microglia, myocytes and fibroblasts were added to the primary motor neurons, there was no effect on survival (Nagai et al., 2007). However, when SOD1^G37R^ variant was selectively deleted in mouse astrocytes *in vivo*, disease onset was not altered, but instead astrocyte-specific deletion of SOD1^G37R^ delayed microglial activation and significantly slowed later disease progression (Yamanaka et al., 2008).

More recently, the knockout of pro-inflammatory cytokines, IL-1α, TNFα and C1q, was found to significantly delay the death of motor neurons in the SOD1^G93A^ mouse model (Guttenplan et al., 2020). In these IL-1α^−/−^ TNFα^−/−^ C1q^−/−^ SOD1^G93A^ mice, lower levels of the microglia-mediated reactive astrocyte marker *C3*, identified in Liddelow et al. (2017), was observed (Guttenplan et al., 2020). Additionally, these triple knockout SOD1^G93A^ mice had a 50% lifespan extension compared to strain matched SOD1^G93A^ mice (Guttenplan et al., 2020). Together these studies provide evidence that astrocytes expressing ALS-linked SOD1 variants have the ability to elicit direct and indirect toxic effects selective to motor neurons, while exacerbating microglial activation and disease progression.

### 2.2 Frontotemporal dementia-associated proteinopathy

Frontotemporal dementia is the clinical term used to encompass a heterogeneous group of disorders associated with progressive behavioral and personality changes, and/or impairments in language and executive function (Bang et al., 2015; Pottier et al., 2016; Woollacott and Rohrer, 2016). FTD is the clinical syndrome resulting from frontotemporal lobar degeneration (FTLD) which can be caused by several different molecular pathologies. There are both familial and sporadic forms of FTD, with the most common variants discovered in genes encoding for the microtubule-associated protein Tau (*MAPT*; FTLD-TAU), progranulin (*GRN;* FTLD-TDP), and *C9orf72* (FTLD-TDP) (Mackenzie and Neumann, 2016).

#### 2.2.1 Tau inclusions

Tau is encoded by the gene *MAPT* and due to alternative splicing in exons 2, 3, and 10, six isoforms of the protein are produced. These isoforms make-up various combinations of three or four microtubule-binding repeat-regions in the C-terminal, and zero to two inserts in the N-terminal; resulting in 0N3R, 1N3R, 2N3R, 0N4R, 1N4R and 2N4R tau species (Buée et al., 2000). It is expected that the isoforms have various specific functions, but the primary function of tau is to promote the assembly of microtubules (Buée et al., 2000). Although tau isoforms are expressed differentially among neuronal subpopulations, tau is not expressed in astrocytes under physiological conditions (Muller et al., 1997).

Neurons are the primary cell type affected by FTLD-TAU, but astrocytes have been identified as a contributor of disease pathology. There is evidence indicating that astrocytic degeneration is associated with the degree of tissue atrophy in the frontal and temporal lobes in FTLD (Broe et al., 2004). FTLD-TAU accounts for 36%–50% of all FTLD cases with Pick’s disease, corticobasal degeneration, and progressive supranuclear palsy as the most common subtypes (Bang et al., 2015). Tufted astrocytes and astrocytic plaques (see Figure 1) are common pathologies in FTLD-TAU (Bang et al., 2015). When iPSC-derived astrocytes expressing the *MAPT* ^N279K^ variant were co-cultured with control neurons, there was a direct toxic effect decreasing neuronal survival during a rotenone-induced oxidative stress assay (Hallmann et al., 2017). Furthermore, these *MAPT* ^N279K^ FTD astrocytes displayed increased cell death and astrogliosis, defined by hypertrophy and increased glial fibrillary acidic protein (GFAP) levels (Hallmann et al., 2017). These results suggest that the *MAPT* ^N279K^ variant elicited a detrimental reactive astrocyte transformation that mediated an astrocyte cell-autonomous and non-cell autonomous neuron pathology. It is important to note that tau-related inclusions overlap with Alzheimer’s disease and enact similar astrocyte pathology, which is further discussed below.

### 2.3 Overlapping amyotrophic lateral sclerosis and frontotemporal dementia proteinopathies

#### 2.3.1 C9orf72-associated dipeptide repeat proteins

The *C9orf72* HRE variant is responsible for 30%–50% of familial ALS cases, 10% of sALS cases, and up to 50% of FTD cases (DeJesus-Hernandez et al., 2011; Renton et al., 2011; Cooper-Knock et al., 2015). The discovery of this variant provided the strongest genetic evidence to date of the significant overlap between ALS and FTD. In addition to TDP-43 pathology (see below), there are three primary non-exclusive mechanisms hypothesized to cause *C9orf72*-ALS/FTD that necessitate the cooperation of both toxic loss and gain-of-functions. The repeat expansion suppresses the production of C9orf72 protein by inhibiting transcription, causing haploinsufficiency and loss-of function of the C9orf72 protein (DeJesus-Hernandez et al., 2011; Renton et al., 2011). The toxic gain-of-function from sense and antisense *C9orf72* repeat RNA, generated by bidirectional transcription, form sense and antisense HRE-RNA foci (DeJesus-Hernandez et al., 2011; Donnelly et al., 2013). The secondary structures of the repeat RNA, G-quadruplexes and DNA/RNA hairpins, bind and sequester RNA-binding proteins and cause DNA damage, respectively (Haeusler et al., 2014). Protein products of the repeat RNA, dipeptide repeat proteins (DPRs), are generated *via* repeat-associated non-ATG (RAN) translation forming five species: poly-GA, poly-GP, poly-GR, poly-PA and poly-PR (Ash et al., 2013; Tran et al., 2015). DPRs are aggregation prone and are thought to have some level of pathogenic effect. However, DPR pathology does not coincide with TDP-43 pathology and neurodegeneration observed in these patients. Nucleocytoplasmic trafficking deficits have also been associated with *C9orf72* pathogenesis (Zhang et al., 2015; Boeynaems et al., 2016; Moore et al., 2020). These pathological features of the disease have been observed in spinal cord, motor, and frontal cortex neurons of *C9orf72*-ALS/FTD patient postmortem tissue (Murray et al., 2011; Mackenzie et al., 2015; DeJesus-Hernandez et al., 2017).

In *C9orf72*-ALS, few studies have described a cell-autonomous astrocyte pathology and an astrocyte-mediated non-cell autonomous motor neuron pathology (Madill et al., 2017; Allen et al., 2019; Birger et al., 2019; Taga et al., 2019; Varcianna et al., 2019; Zhao et al., 2020). One study demonstrated that *C9orf72*-ALS human induced pluripotent stem cell (iPSC)-derived spinal cord astrocytes develop RNA foci and DPR pathology (Zhao et al., 2020). DPRs have led to mis-splicing of the *SLC1A2* gene causing dysfunction of EAAT2 (Lin et al., 1998; Kwon et al., 2014). Consequently, DPR pathology in astrocytes reduces their ability to uptake glutamate from their surroundings, leading to glutamate excitotoxicity in motor neurons already made vulnerable by C9orf72 haploinsufficiency (Shi et al., 2018). In co-culture, *C9orf72*-ALS astrocytes with confirmed RNA foci and poly-GP presence, triggered adverse effects on the electrophysiological properties of motor neurons (Zhao et al., 2020). Additionally, it has been found that DPRs can be transmitted from neurons to astrocytes through both contact dependent and independent mechanisms (Westergard et al., 2016). The transmission of different DPR species leads to nuclear and cytoplasmic aggregates in astrocytes co-cultured with DPR transfected neurons (Westergard et al., 2016).

Furthermore, when exposed to *C9orf72*-ALS astrocyte derived extracellular vesicles (EVs) and conditioned media, motor neurons experienced a 50%–70% increase in death compared to control astrocyte EVs and conditioned media (Varcianna et al., 2019). In this same study, *C9orf72*-ALS astrocytes were observed to secrete fewer EVs compared to controls (Varcianna et al., 2019). This contrasts studies describing astrocytes expressing SOD1 variants having an increase in EV secretion, therefore suggesting a characteristic difference between SOD1 and *C9orf72* ALS astrocytes (Basso et al., 2013; Varcianna et al., 2019). Together, these studies indicate a role for C9orf72 astrocytes in the neurodegeneration of motor neurons observed in *C9orf72*-ALS/FTD; however, there is little to no information on the role of astrocytes in the neurodegeneration of cortical neurons leading to dementia symptoms in this disease spectrum.

#### 2.3.2 TDP-43 proteinopathy–Nuclear depletion and cytoplasmic mislocalization

The hallmark pathology of approximately 97% of ALS cases and 50% of FTD cases is the simultaneous nuclear depletion and cytoplasmic accumulation of TDP-43 (TAR-DNA binding protein 43), encoded by the *TARDBP* gene (Arai et al., 2006; Neumann et al., 2006). TDP-43 is an RNA and DNA binding protein, serving multiple functions in the regulation of gene expression at both the transcription and translation levels (Ratti and Buratti, 2016). Similar to other heterogeneous nuclear ribonucleoproteins (hnRNPs), TDP-43 consists of an N-terminus region followed by two RNA recognition motifs (RRMs) that bind RNA and DNA, and a prion-like domain containing C-terminus that aids in its ability to participate in protein-protein binding, phase separation and aggregation. Predominantly a nuclear protein, TDP-43 can shuttle to and from the cytoplasm *via* active and passive transport under physiological conditions. However, the aberrant nuclear depletion and cytoplasmic mislocalization of TDP-43 causes two toxic mechanisms—a toxic loss-of-function in the nucleus and toxic gain-of-function in the cytoplasm that work in conjunction (Tziortzouda et al., 2021). The resulting pathology in affected neurons and glia are cytoplasmic inclusions containing TDP-43, phosphorylated TDP-43 (pTDP-43), and cleaved C-terminal fragments. This pathology primarily occurs in neurons in the spinal cord, frontal, temporal and primary cortices, as well as subcortical regions, such as the hippocampus, basal ganglia, amygdala, thalamus and regions within the brainstem (Arai et al., 2006; Neumann et al., 2006; Murray et al., 2011; Mackenzie et al., 2015). TDP-43 inclusions are found in the cytoplasm and radiating processes of astrocytes (see Figure 1), but are more common in oligodendrocytes in the spinal cord and some cortical regions (Nishihira et al., 2008; Cooper-Knock et al., 2012; Heo et al., 2022).

The achievement of TDP-43 pathology *in vitro* and *in vivo* models most often requires either biological stressors or overexpression of specific TDP-43 variants. Astrocytic models require TDP-43 (full length, phosphorylated or the C-terminal fragment) to be selectively overexpressed or seeded into astrocytes (Serio et al., 2013; Hebron et al., 2014; Heyburn et al., 2016; Peng et al., 2020; Velebit et al., 2020). In a recent study investigating TDP-43 proteinopathy in sALS, hiPSC-derived motor neurons and astrocytes were exposed to seeded pathological TDP-43 aggregates from serially passaged sALS postmortem tissue (Smethurst et al., 2020). Through this seeding model, iPSC-motor neurons were observed to readily transfer TDP-43 aggregates to iPSC-astrocytes and *vice versa*. However, TDP-43 aggregates in iPSC-astrocytes were cleared more readily and there were less aggregates transferred from astrocytes to motor neurons (Smethurst et al., 2020). This study suggests a cell-type specific vulnerability and protein clearance of TDP-43, whereby astrocytes are less vulnerable and more efficient at clearing TDP-43 than motor neurons.

Another study, using a conditional TDP-43 knockdown mouse model to investigate the effects of nuclear depletion of TDP-43 in spinal cord astrocytes, revealed enhanced GFAP immunoreactivity and a molecular signature resembling that of the “A1-astrocytes” identified in Liddelow et al. (2017) (Peng et al., 2020). These TDP-43 depleted astrocyte mice presented with motor deficits, upregulation of C1 complement expression in microglia, and reduction of mature oligodendrocytes without directly affecting motor neuron survival (Peng et al., 2020). This indicates that nuclear loss-of-function of TDP-43 in affected astrocytes has both a cell autonomous effect and a non-cell autonomous effect on other glial types. Furthermore, a study that transfected primary rat cortical astrocytes with the C-terminal fragment of TDP-43, found that affected cells developed cytoplasmic TDP-43 inclusions and nuclear depletion of endogenous TDP-43 (Velebit et al., 2020). This is consistent with findings in neurons and cell lines where expression of the C-terminal fragment affects endogenous TDP-43 nucleocytoplasmic trafficking (Geser et al., 2008; Winton et al., 2008). Transfected astrocytes with TDP-43 cytoplasmic inclusions also presented with a stress state that involved an increase in lipid droplets, signifying an impaired lipid metabolism and disruptions in ß-adrenergic mediated aerobic glycolysis and lactate production (Velebit et al., 2020). This study further supports the idea that astrocytes affected by TDP-43 pathology in ALS and FTD may undergo a reactive transformation that disables an astrocyte’s ability to metabolically support surrounding neurons.

Given the dominant role of TDP-43 in ALS pathogenesis, ALS-linked TDP-43 variants have also been studied in astrocytes. In iPSC-derived astrocytes, carrying the TDP-43^M337V^ variant, cells displayed cytoplasmic mislocalization of TDP-43, but not simultaneous nuclear depletion (Serio et al., 2013). Motor neurons with the same TDP-43^M337V^ variant presented with high levels of insoluble TDP-43 (Bilican et al., 2012), whereas astrocytes did not (Serio et al., 2013). Notably, this suggests a cell-type specific function of TDP-43 between neurons and astrocytes. *In vivo*, the same TDP-43^M337V^ variant expressed only in rat astrocytes led to the progressive loss of spinal motor neurons, skeletal muscle atrophy, and eventual paralysis (Tong et al., 2013). However, a separate study where the TDP-43^A315T^ variant was overexpressed in astrocytes, found no motor neuron death in either an *in vitro* or transplant mouse model (Haidet-Phillips et al., 2013). The lack of consensus among these studies regarding whether astrocytes harboring a TDP-43 disease related-variant can cause motor neuron death may be due to variant-specific astrocyte-associated TDP-43 dysfunction. Additionally, variations in protein processing responses between cell types and models could also account for these differences.

#### 2.3.3 FUS proteinopathy

Another RNA binding protein commonly associated with both ALS and FTD is the fused in sarcoma (FUS) protein. This predominantly nuclear protein also has a large role in regulating RNA metabolism events (Lagier-Tourenne and Cleveland, 2009; Dormann et al., 2010). Similar to TDP-43 pathology, FUS pathology is characterized by a simultaneous nuclear depletion and cytoplasmic mislocalization (Deng et al., 2010; Mackenzie et al., 2011). However, ALS and FTD cases with FUS inclusions do not present with TDP-43 inclusions; *vice versa* for cases with TDP-43 inclusions (Ling et al., 2013). ALS and FTD related variants in FUS lead to a dysfunctional protein. The ubiquitous knock down and conditional knock out of FUS in mice does not lead to ALS related motor deficits, but rather behavioral impairments (Kino et al., 2015; Udagawa et al., 2015). FUS inclusions are observed in neurons and glia in human and model tissue (see Figure 1), with a specific set of variants causing wide-spread FUS pathology triggering cell death (Kwiatkowski et al., 2009; Blair et al., 2010; Hewitt et al., 2010; Suzuki et al., 2012). Primary astrocytes transduced with FUS^R521G^ variant, presented with cytoplasmic mislocalization in 90% of the cultured cells and a substantial increase in GFAP protein expression (Kia et al., 2018). Additionally, motor neuron death occurred in cultures treated with conditioned media from the FUS^R521G^ astrocytes (Kia et al., 2018). This study indicated that the presence of FUS^R521G^ in astrocytes was not detrimental to astrocytes, rather it caused a toxic phenotype that triggered motor neuron death through secreted factors like TNFα (Kia et al., 2018).

#### 2.3.4 ADAR2 nucleocytoplasmic mislocalization

The RNA binding protein adenosine deaminase acting on RNA 2 (ADAR2), was recently found to also undergo nucleocytoplasmic mislocalization (Moore et al., 2019). ADAR2 is a member of the ADAR family whose primary role is to deaminate adenosine residues, specifically in double-stranded RNA (Melcher et al., 1995; O'Connell et al., 1995). This catalytic process converts a single adenosine to an inosine resulting in A-to-I editing (Melcher et al., 1995; Hwang et al., 2016). In *C9orf72*-ALS/FTD human tissue from the motor cortex and spinal cord, ADAR2 was found to mislocalize and form cytoplasmic accumulations, and co-localize with TDP-43 inclusions in the spinal cord (Moore et al., 2019). Nucleocytoplasmic mislocalization of ADAR2 was also observed in *C9orf72*-ALS/FTD hiPSC-motor neurons and in an AAV9-(G_4_C_2_)_149_-transduced WT mouse model (Moore et al., 2019). Substantial RNA editing aberrations associated with A-to-I editing were identified in spinal cord, cerebellum, motor and frontal cortices from *C9orf72*-ALS/FTD patients (Moore et al., 2019). Although a rigorous characterization of ADAR2 mislocalization and its transcriptional consequences in motor neurons were performed, there is no evidence yet supporting the mislocalization of ADAR2 in astrocytes or any other glial cells.

Collectively, these studies suggest astrocytes have a critical involvement in the degeneration of neurons through the loss of supportive and/or gain of toxic functions in various genetic subtypes of ALS and FTD. It is important to note that a *C3* ^*+*^ reactive astrocyte transformation has been observed in C9orf72, SOD1 and sALS human tissue samples in the spinal cord and motor cortex, indicating a neuroinflammatory astrogliosis as a common pathology in ALS (Guttenplan et al., 2020). This is further supported by several studies utilizing RNA-sequencing data from ALS mouse models and iPSC-derived astrocytes, indicating that ALS astrocytes undergo a cell autonomous reactive transformation and pro-inflammatory profile in early disease stages (Ziff et al., 2021; Taha et al., 2022). However, the notion that astrocytes are directly the cause of motor neuron death is unlikely, instead it seems that diseased astrocytes exacerbate the vulnerable condition of diseased neurons. Whether this aggravation is due to a compensatory mechanism initiated by a loss of homeostatic functions and/or a toxic reactivity state is unclear. What is clear is that astrocytes do develop similar protein accumulations to neurons such as TDP-43 cytoplasmic inclusions, SOD1 nuclear accumulations, and DPRs. Nevertheless, the incidence of these protein accumulations in astrocytes is reduced compared to neurons, which suggests astrocytic clearance is either more efficient or not completely affected by underlying disease mechanisms (Yoshiyama et al., 2003; Forman et al., 2005; Serio et al., 2013; Westergard et al., 2016; Kia et al., 2018; Shi et al., 2018; Wang and Ye, 2021).

## 3 Other neurodegenerative diseases

### 3.1 Alzheimer’s disease

Alzheimer’s disease (AD) is the most common neurodegenerative disease-causing dementia. The onset of disease for a majority of patients is after the age of 65 (late-onset AD; LOAD), whereas a smaller population of patients will experience clinical symptoms before the age of 65 (early onset AD; EOAD) (Long and Holtzman, 2019). Pathogenesis of disease can be inherited and caused by specific genetic variants in genes *APP*, *PSEN1* and *PSEN2* (St George-Hyslop et al., 1987; Schellenberg et al., 1992; Goate et al., 1991; Sherrington et al., 1995)*,* and with higher risk of disease development in carriers of the apolipoprotein E (*APOE*) ε4 allele (Pericak-Vance et al., 1988; Corder et al., 1993; Strittmatter et al., 1993). These are usually associated with faster progressing patients compared to sporadic AD cases. AD patients suffer from a set of heterogenous clinical and preclinical symptoms such as deficits in learning and memory, attention, executive function, language, visuospatial function, and behavioral impairments (Jack et al., 2018). The hallmark pathologies of AD are the extracellular deposits of amyloid-ß that form diffuse and neuritic plaques, and the intracytoplasmic neurofibrillary tangles (NFTs) that consist of aggregated hyperphosphorylated tau protein. Additionally, TDP-43 pathology, similar to what is observed in ALS and FTD, has also been found to occur in various brain regions of AD patients. There is substantial evidence indicating a localized role of reactive astrocytes in AD pathogenesis (Liddelow et al., 2017). Whereby these cells undergo both gains and losses of functions that are dependent on their given subtype and regional pathogenic load (Sadick et al., 2022). Here, we discuss AD-associated amyloid-ß plaques and neurofibrillary tau tangles and their proximity and distribution within astrocytes. Then, we will discuss AD-associated TDP-43 pathology and potential astrocytic occurrence, briefly review how it compares to FTD-tau related pathology, and incidence with other AD proteinopathies.

#### 3.1.1 Amyloid-ß plaques

Amyloid-ß (Aß) is the product of the proteolytic cleavage of the amyloid precursor protein (APP). Cleaved sequentially by ß-secretase and γ-secretase, the Aß protein ranges from 40–42 amino acids in length (Hardy and Allsop, 1991). Identification of genetic variants in the *APP*, *PSEN1* and *PSEN2* genes associated with familial AD, were found to favor the longer Aß_42_ peptide and enhance its production (Chartier-Harlin et al., 1991; Goate et al., 1991; Murrell et al., 1991; Citron et al., 1992; Hendriks et al., 1992; Mullan et al., 1992; Cai et al., 1993; Scheuner et al., 1996). These findings resulted in the conception of the controversial “Amyloid Cascade Hypothesis” that asserts the imbalance of Aß_42_ production and clearance is the cause of AD (Beyreuther and Masters, 1991; Hardy and Allsop, 1991; Hardy and Higgins, 1992). The pathological distribution of Aß begins in pre-clinical stages, where deposits can be found in neocortical areas of high metabolic demand, then extending into the allocortex and brainstem whereby patients have transitioned into clinical stages (Thal et al., 2000; Buckner et al., 2005). However, failures in therapeutics against Aß have resulted in a refocus from improving the clearance of Aß plaques to also improving the clearance of NFTs and other misfolded aggregate prone proteins, and alleviating neuroinflammation—all of which are believed to contribute to neuronal death and the clinical manifestations of AD.

Surrounding Aß plaques are activated microglia and astrocytes (see Figure 1) (Pike et al., 1995; Bates et al., 2002). Astrocytes have been shown to be important mediators of amyloid-ß clearance (Thal et al., 2000). Specifically, astrocytes can internalize Aß_1-42_ *in vitro*, with aggregate size and conformation limiting astrocytic Aß uptake (Nielsen et al., 2010). In a 2010 study, primary adult human astrocytes from the temporal lobe were shown to internalize smaller size Aß_1-42_ oligomers more efficiently than fibrillar Aß_1-42_ species (Nielsen et al., 2010). Furthermore, amyloid associated proteins, like ApoE, significantly altered the astrocytic uptake of oligomeric Aß, suggesting a mechanism that could ultimately effect clearance and deposition at different disease stages (Nielsen et al., 2010). Reactive astrocytes found in postmortem AD tissue of the entorhinal cortex have a significant Aß load (Nagele et al., 2003), however astrocytes with large phagocytic burden become inefficient at degradation of cell debris (Loov et al., 2012). One study found that Aß accumulation in astrocytes resulted in enlarged endosomes, where engulfed Aß_42_ was stored instead of being actively degraded (Söllvander et al., 2016). These intracellular Aß_42_ accumulations further caused endosomal/lysosomal defects, which led to microvesicle-induced apoptosis of neurons (Söllvander et al., 2016).

A recent study aimed at characterizing activation states within AD pathogenesis used single-nuclei RNA-sequencing to identify differences in astrocyte states in the hippocampus of the 5xFAD transgenic AD mouse model (Habib et al., 2020). This mouse model expresses five familial AD-linked variants in the human *APP* and *PS1* transgenes, causing severe extracellular amyloid deposition, synaptic and cortical neuron loss in layer V and the subiculum, and impaired spatial and working memory deficits (Oakley et al., 2006). A continuous range of astrocyte profiles were identified and divided into six transcriptional sub-clusters. Both AD and WT groups had clusters ranging from a homeostatic Gfap-low state, to an intermediate state, into a Gfap-high state (Habib et al., 2020). The AD group had an additional Gfap-high cluster that was designated as the diseased-associated astrocyte (DAA) cluster and an intermediate cluster between the Gfap-high and DAA clusters, described as a transitional DAA state. The DAAs were associated with different molecular pathways involved in amyloid accumulation, metabolism and clearance, and the proteolytic processing of APP. The authors determined that the DAAs were only present in the hippocampus of AD mice using co-staining of GFAP^+^SERPINA3N^+^VIM^+^ astrocytes. DAAs were also found to be adjacent to Aß plaques, appearing before the onset of cognitive decline and continuing to develop in parallel with disease progression in the AD model. The DAAs were found to develop from homeostatic Gfap-cells in WT mice, who acquired these with age, suggesting a similar phenotypic DAA-like stage that also occurs in normal aging. In human postmortem tissue, DAA-like cells were identified with higher frequency in AD patients along a spectrum with the intermediate transitional DAA cluster, which suggests a dynamic activation process in AD. This study supports the notion that astrocytes take on pathogenic changes with a role in early stages of AD disease pathogenesis.

#### 3.1.2 Neurofibrillary tau tangles

In the pathological context of AD, the 3R and 4R ratio of tau isoforms is disturbed, leading to aggregation and NFT formation. Tau pathology begins in the entorhinal cortex, spreading to limbic regions and then finally to the neocortex (Braak and Braak, 1991; Braak et al., 2011). Because tau is not expressed in astrocytes (Muller et al., 1997), the source of tau in astrocytic accumulations in AD is unknown. Nevertheless, a recent study described tau accumulation in hilar astrocytes (see Figure 1) of the dentate gyrus in patients (Richetin et al., 2020). The presence of 3R tau^+^ inclusions were more prevalent in astrocytes in the hippocampus and dentate gyrus compared to controls, with incidence increasing with disease state (Richetin et al., 2020). The occurrence of 3R tau^+^ inclusions were exacerbated when hyperphosphorylated tau and Aß plaques were observed, which correlated with synaptic alterations. Astrocytic 4R tau^+^ inclusions were present, but only in cases without hyperphosphorylated tau and Aß plaques. Furthermore, when 1N3R tau was overexpressed in astrocytes *in vivo*, these cells experienced impaired mitochondrial motility and function, and decreased adult neurogenesis and inhibitory synapses, ultimately contributing to impaired spatial memory in the animals (Richetin et al., 2020). In a recent study examining the transmission of tau aggregates released by neurons and its effects on receptive astrocytes, it was observed that filamentous tau aggregates bind to the integrin receptor complex ITG⍺V/ß1 located on immune-panned primary mouse astrocytes (Wang and Ye, 2021). It was observed that this integrin complex facilitated the uptake of tau fibrils while also regulating tau fibril-induced inflammation and conversion to a reactive astrocyte state similar to that of an “A1 astrocyte” (Wang and Ye, 2021). The authors proposed that astrocytic integrin receptors act as a sensing mechanism for extracellular misfolded proteins that help with clearance. However, when overburdened by these extracellular misfolded proteins astrocytes then shift towards an inflammatory reactivity state. Providing further evidence that the overexpression or presence of tau in astrocytes may lead to disruptions in their trafficking network of cellular cargo leading to degeneration of not only neurons, but also astrocytes themselves (Yoshiyama et al., 2003; Forman et al., 2005). The role of astrocytes in tauopathies, other than AD, has been extensively reviewed in Kovacs (2020).

#### 3.1.3 TDP-43 inclusions

In 57% of AD cases there is evidence of TDP-43 pathology similar to what is observed in ALS and FTD (McAleese et al., 2017). Recently updated staging schemes describe TDP-43 pathology occurring first within limbic brain regions such as the amygdala, hippocampal formation and entorhinal cortex, with early distribution in the occipitotemporal cortex and ventral striatum (stages 1–3), and finally extending into neocortical areas such as the mid-temporal and mid-frontal cortex, as well as the basal ganglia (stages 4 and 5/6) (Josephs et al., 2016; Nag et al., 2018). TDP-43 inclusions are predominantly found in neurons, but there are reports of glial cytoplasmic inclusions (GCI) of TDP-43 in AD tissue (see Figure 1). However, GCIs are found only in transferrin-positive oligodendrocytes (Higashi et al., 2007).

A recent study examined whether TDP-43 in AD represents a coexistence of AD and FTD-TDP or if the two TDP-43 pathologies are discrete. They identified distinct molecular patterns of TDP-43 species in AD human tissue of hippocampus, entorhinal, frontal, temporal and occipital cortex, amygdala (Tome et al., 2020). They found a range of TDP-43 inclusion patterns from the absence of inclusions to inclusions only positive for pTDP-43 (serine 409/410), and inclusions positive for pTDP-43 (serines 403/404 and 409/410) and full length TDP-43 (N and C-terminal domains), similar to that of FTLD-TDP. About 20% of AD cases with only pTDP-43 inclusions, presented with language deficits, and about 36% of AD cases positive for both pTDP-43 and full length TDP-43 experienced FTD-like behavioral or language problems. This further supported the hypothesis that distinct phosphorylation sites have an impact on the type of TDP-43 pathology and its relation to FTD-like symptoms observed in AD. However, glial TDP-43 inclusions were not individually assessed, but were considered in the classification of samples.

Limbic-predominant age-associated TDP-43 encephalopathy-related neuropathological changes (LATE-NC), is a newly defined TDP-43 related proteinopathy with inclusions primarily in limbic regions of the brain (Nelson et al., 2019). LATE-NC affects up to 50% of individuals past the age of 80 years old and individuals with or without AD (Nelson et al., 2019). LATE-NC clinically manifests with amnestic cognitive syndrome affecting emotional lability and global cognitive function, which is independent of co-existing pathologies (Nelson et al., 2019). TDP-43 proteinopathy extends from the amygdala to the hippocampus, then to the middle frontal gyrus; this distribution can occur with or without hippocampal sclerosis (Nelson et al., 2019). Individuals with LOAD with TDP-43 pathology in limbic regions are now recognized as having simultaneous LATE-NC. A diagnosis of ALS and/or FTLD-TDP excludes a diagnosis of LATE-NC. Unlike ALS and FTD, there are rare mentions of glial TDP-43 inclusions associated with LATE, however there is no clarification on glial cell type (Hunter et al., 2020).

Finally, there are a handful of studies implicating TDP-43 interactions with Aß and tau. Studies investigating the relationship between Aß and TDP-43 pathology in AD have found that Aß_42_ expression *in vivo* upregulated endogenous TDP-43 and caused cytosolic pTDP-43 inclusions (Herman et al., 2011; Herman et al., 2012). Using an AD mouse harboring *APP* and *PS1* variants (APP/PS1 model), the injection of TDP-43 oligomers increased Aß plaques and further impaired spatial memory (Shih et al., 2020). The relationship between tau and TDP-43 has indicated that TDP-43 has a role in assisting with the *MAPT* mRNA splicing leading to a decrease of 4R isoforms by exclusion of exon 10 (Gu et al., 2017). Furthermore, ALS/FTD related TDP-43 variants led to inclusion of exon 10, resulting in an increase and disruption of the 3R:4R ratio (Gu et al., 2017). Specificity of phosphorylation sites on TDP-43 dictate its ability to suppress exon 10 in *MAPT* mRNA (Gu et al., 2018). Additionally, TDP-43 and tau have been shown to have a collaborative seeding process in the brain whereby TDP-43 oligomers could serve as a nucleation site for tau aggregates (Montalbano et al., 2020). However, these interactions have not been identified or studied in astrocytes or any other glia.

The role of astrocytes in AD is ultimately important to maintain the integrity of neurons; however, there is evidence that AD pathology has an early and eventual impact on astrocytic integrity caused by a reactive transformation. Both Aß and tau pathology have been observed in relation to astrocytes in both models of AD and AD human tissue. In a recent study investigating how the characteristic AD proteinopathies influence astrocytes, TRAP-seq data from mouse models of tauopathy and ß-amyloidopathy revealed that neither of these proteinopathies triggered “A1” or “A2” reactivity profiles in these mice, but rather model specific deleterious and adaptive-protective profiles (Jiwaji et al., 2022). Specifically, these pathologies induced activation of inflammatory pathways, alongside protein degradation pathways, all the while exhibiting an induction of a set of genes normally elevated in aged mouse brains (Jiwaji et al., 2022). Thus, furthering the argument that astrocyte reactivity is context and disease dependent. TDP-43 pathology has also been observed in glia, but the identification of TDP-43 in an AD context in astrocytes has yet to be properly described. There is much more research needed in describing AD-related pathology in astrocytes and identifying astrocyte specific perturbations that occur due to the accruement of Aß, tau, and TDP-43 pathologies.

### 3.2 Huntington’s disease

Huntington’s disease (HD) is an autosomal dominant neurodegenerative disease caused by the expansion of the CAG repeat in the huntingtin (HTT) gene, resulting in a poly-glutamine tract in the translated protein. HD onset commonly occurs around 45 years of age, with aggressive progression of involuntary choreiform movements (repetitive, rapid, jerky movements); in addition, patients can experience behavioral, psychiatric, and dementia like symptoms (Ghosh and Tabrizi, 2015). However, there are cases where motor symptom onset can begin in childhood (Ghosh and Tabrizi, 2015). Patients generally have 36 or more CAG repeat units, with longer CAG expansions associated with earlier disease onset (Andrew et al., 1993). Much of the neurodegeneration in HD occurs in striatal medium spiny neurons (MSNs), as these are more vulnerable to the HTT expansion variant’s cellular disruptions. However, there is also substantial cellular disruption and death in the cortex, accompanied by progressive reactive astrogliosis (Ghosh and Tabrizi, 2015). HD neuropathology is measured on a graded scale of 0–4 that is dependent on the degree of striatal atrophy and astrocyte reactivity (Rüb et al., 2015; Khakh et al., 2017). The major pathological hallmark of HD are large inclusions of expanded HTT fibrils in the nucleus and cytoplasm of cells (see Figure 1) (DiFiglia et al., 1997; Cooper et al., 1998). Additionally, there have been occurrences of a co-pathology with TDP-43 inclusions in neurons (Schwab et al., 2008). The large expanded HTT inclusions are not only found in neurons, but also at comparable levels in cortical and striatal astrocytes of HD patients and models (Shin et al., 2005; Faideau et al., 2010; Tong et al., 2014; Jansen et al., 2017). Expanded HTT in astrocytes occurs in grade 0 of HD pathogenesis, suggesting that astrocytes may contribute to disease progression (Faideau et al., 2010). The physiological function of the huntingtin protein is still relatively undetermined. Information about the protein sequence, posttranslational modifications and regional expression patterns implicate the protein in CNS developmental processes, cell survival, vesicle transport, and synaptic maintenance (De Souza and Leavitt, 2015).

Evidence from human and HD murine model tissue display expanded HTT accumulations in striatal astrocytes, indicative of an astrocytic role in HD pathogenesis (Shin et al., 2005; Faideau et al., 2010; Tong et al., 2014). It has been observed that astrocytic expression of expanded HTT has the ability to reduce the expression of both Glt-1, the murine homolog of the astrocyte specific glutamate receptor EAAT2, and Kir4.1, a potassium ion channel that aids in extracellular K^+^ buffering (Lievens et al., 2001; Behrens et al., 2002; Shin et al., 2005; Faideau et al., 2010; Tong et al., 2014; Jiang et al., 2016). This reduction allows for an increase in extracellular glutamate and K^+^, increasing the excitability of MSNs and leaving them susceptible to excitotoxicity. In HD affected regions, astrocytes exhibit a reactive state defined by an increase in GFAP expression (Selkoe et al., 1982). When expanded HTT is expressed selectively in astrocytes, these cells undergo morphological changes and hypertrophy with these changes being greater in the putamen compared to the caudate nucleus (Faideau et al., 2010), suggestive of regional differences of astrocyte HD pathology. When striatal astrocytes expressing expanded HTT were co-cultured with striatal neurons there was an increase in neuron death (Aizawa et al., 2016). However, when expanded HTT is expressed only in cortical neurons *in vivo*, these mice do not develop neuropathology (Gu et al., 2005).

Two HD mice models are widely used, the transgenic R6/2, a model of aggressive disease progression mimicking juvenile HD (Mangiarini et al., 1996), and the knock-in Q175, a model with less aggressive progression mimicking adult-onset HD (Menalled et al., 2003). The amount of nuclear expanded HTT inclusions in astrocytes increases as both models develop symptoms and pathology (Tong et al., 2014). The astrocytes in the striatum have electrophysiological defects, such as depolarized membrane potentials and lower membrane conductance, attributed to reduced expression of Kir4.1 (Tong et al., 2014). By increasing astrocytic Kir4.1 the motor deficits and longevity of both models improved, and even more interestingly the expression of the glutamate transporter Glt-1 increased (Tong et al., 2014).

In a recent study examining the molecular signatures of astrocytes in HD, again the two HD mice models, R6/2 and Q175, were used in conjunction with human HD postmortem tissue to assess astrocyte specific gene and protein expression (Diaz-Castro et al., 2019). There were a core set of gene expression changes in both HD models and human tissue samples that confirmed altered functions in Ca^2+^ signaling, G-protein coupled receptors (GPCRs), and neurotransmitter regulation. The mice were assessed at three different ages, and the functions of the common dysregulated genes were similar at early and late stages. To further support that data, astrocyte reactivity was identified in human tissue samples from patients at grade 3 and altered astrocyte gene expression was observed as early as grade 0. This is indicative of early astrocyte dysfunction in HD that persists throughout disease progression. Additionally, there was little evidence indicating an “A1 neurotoxic” astrocyte signature, further supporting the idea that astrocyte reactivity is context specific.

To explore the notion of context-specific and region-specific astrocyte differences, 14 experimental perturbations relevant to the contribution of striatal astrocytes in HD were selected and executed in the R6/2 model (Yu et al., 2020). At the level of genes, pathways and upstream transcriptional signaling regulators, the molecular responses of striatal astrocytes were found to be context dependent. With this data, the authors identified that activation of GPCR signaling in striatal astrocytes could correct HD related astrocyte alterations that effect Ca^2+^ signaling and astrocyte territory size, in addition to an enhancement of neuroimmune responses. Striatal astrocyte GPCR activation corrected behavioral deficits in the R6/2 model, effecting anxiety-related behaviors and motor function. Interestingly, there was also an observed increased expression of thrombospondin 1, a synaptogenic astrocyte-secreted protein important for the formation of synapses.

Together these studies suggest the disruption of essential functions of astrocytes and astrocyte-neuron interactions having a large role in HD pathogenesis. A recent study employing single-nucleus RNA-sequencing of cingulate cortex from grade III/IV HD patients found three distinct reactivity states among the astrocyte cluster (Al-Dalahmah et al., 2020). These reactivity states ranged from low, intermediate and high GFAP expression, while all had decreased expression of glutamate transporters *SLC1A2* and *SLC1A3* (Al-Dalahmah et al., 2020). The authors report HTT aggregates in a small population of astrocytes in the cingulate cortex from cases used for the snRNA-sequencing, and further suggest that reactive transformation of the astrocytes is secondary to neuronal loss, but exacerbated by the reactive nature of the surrounding astrocytes (Al-Dalahmah et al., 2020). Further contributing to the evidence of astrocytic expanded HTT inclusions and a reactive astrocyte transformation in HD, ultimately exacerbating disease pathogenesis.

### 3.3 Parkinson’s disease

Parkinson’s disease (PD) is the second-most common age-related neurodegenerative disease after AD (Wright Willis et al., 2010). Most PD cases are idiopathic, but there are various monogenic mutations that have been linked with disease development. The hallmark pathology of PD is the degeneration of dopaminergic (DA) neurons in the substantia nigra pars compacta (SNpc) and the depletion of dopamine levels in the striatum (Kordower et al., 2013). In addition to the loss of DA neurons, there are intracellular accumulations of misfolded proteins known as Lewy bodies. These inclusions consist of α-synuclein and other associated proteins such as phosphorylated tau and amyloid-ß (Maiti et al., 2017). Diagnosis of PD is based on the presence of at least two of the following clinical symptoms: resting tremor, bradykinesia, muscle rigidity, and postural imbalance or impaired gait (de Lau and Breteler, 2006; Dickson, 2012; Maiti et al., 2017). Additionally, asymmetric symptom onset and good response to levodopa further supports a parkinsonian diagnosis (de Lau and Breteler, 2006). In about half of PD patients there are also executive function deficits mediated by the fronto-striatal pathways, such as attention, mental processing speed, and impairments in language, working memory and impulsivity (Dickson, 2012; Maiti et al., 2017). Here we will discuss what is known about the distribution of α-synuclein inclusions in astrocytes and their effects on astrocyte function. Then we will briefly discuss monogenic mutations that cause parkinsonian phenotypes and their effects on astrocyte function.

The role of astrocytes in PD is still unclear, however there is substantial evidence implicating these cells in disease progression. The gene *SNCA*, encoding the protein α-synuclein is lowly expressed in astrocytes compared to neurons. However, there are studies showing α-synuclein^+^ inclusions in astrocytes of PD patient tissue (Wakabayashi et al., 2000; Braak et al., 2007). Specifically in sporadic PD cases, these α-synuclein^+^ astrocytes are found within the SN, midbrain, amygdala, thalamus, striatum and cerebral cortex (Wakabayashi et al., 2000; Braak et al., 2007). In early stages of disease pathogenesis, these α-synuclein^+^ astrocytes are primarily found in the amygdala, striatum, and thalamic nuclei projecting to the cortex (Braak et al., 2007). In the cortex, these astrocytic inclusions are observed in layers V and VI, with limited prevalence in layers III and IV (Braak et al., 2007). Previous studies suggest that the α-synuclein found in astrocytes are possibly transferred from neurons (see Figure 1) and may be an exogenous pro-inflammatory activator of astrocytes (Lee et al., 2010; Fellner et al., 2013; Rannikko et al., 2015).

In a 2010 study by Lee et al., they observed that SH-SY5Y derived α-synuclein was endocytosed by rodent astrocytes and further concentrated in the astrocytic lysosomes. This direct transmission of “neuronal” α-synuclein to astrocytes also caused an astrocytic inflammatory response and build-up of glial cytoplasmic inclusions (Lee et al., 2010). Further exploring the effect of α-synuclein on astrocytes, a recent study tested different α-synuclein species and how they would induce an astrocytic inflammatory response (Russ et al., 2021). iPSC-derived astrocytes from healthy individuals were treated with TNFα or various α-synuclein species (monomers, oligomers, high molecular weight fibrils). Astrocytes treated with TNFα experienced a pro-inflammatory phenotype characterized by an increase in cytokine release, nuclear localization of NF-κB and altered mitochondrial function, whereas α-synuclein treatment did not. Instead α-synuclein treated astrocytes took on an antigen-presenting phenotype, similar to oligodendrocyte precursor cells in an *in vitro* model of multiple sclerosis (Kirby et al., 2019). Furthermore, only α-synuclein fibrils caused alterations in mitochondrial function, similar to that of TNFα treatment (Russ et al., 2021). Treatment of PD iPSC-astrocytes with α-synuclein resulted in an exacerbated pro-inflammatory response with an increase in secretion of pro-inflammatory cytokines. Co-treatment of TNFα and α-synuclein resulted in a decrease in the effects resulting from TNFα or α-synuclein treatment alone. This suggested to the authors that the astrocytic responses to α-synuclein can be positively altered in the presence of pro-inflammatory cytokines, but negatively altered by degradation resistant α-synuclein aggregate species.

It has been further hypothesized that an increase in α-synuclein concentration can overload the astrocytic lysosomal system, causing α-synuclein accumulations and aggregates that may lead to further deficits in physiological astrocyte function. In a recent study, astrocytes derived from embryonic stem cells were shown to engulf large amounts of α-synuclein (Rostami et al., 2017). These large oligomers were observed to be stored in the trans-golgi region that led to defects in the phagosomal-lysosomal machinery within the astrocytes. Ultimately these astrocytes produced tunneling nanotubes that allowed physical connection to neighboring cells, mediating α-synuclein cell-cell transfer. Additionally, there are reports of deficits in astrocytic glutamate uptake resulting in excitotoxicity similar to what is observed in ALS, which has been thoroughly reviewed (Hindeya Gebreyesus and Gebrehiwot Gebremichael, 2020).

A small percentage of PD cases are attributed to monogenic mutations in *LRRK2*, *Parkin, PINK1* and increased risk of PD with *GBA1* variants (Klein and Westenberger, 2012). The leucine-rich repeat kinase 2 is encoded by the *LRRK2* gene and causes autosomal dominant PD through multiple pathogenic variants with varying penetrance (Roosen and Cookson, 2016; Tolosa et al., 2020). Depending on the mutation, LRRK2-PD can present differently neuropathologically. About 50% of individuals have nigral degeneration, and 65%–80% will present with typical Lewy body pathology (Kalia et al., 2015). Other pathological changes can include non-specific cell loss without signs of proteinopathy, and, interestingly, tau and TDP-43 pathology (Vilas et al., 2018). LRRK2 has been implicated in regulating the autophagy-lysosome pathway. In primary mouse astrocytes, PD-associated mutations within LRRK2 compromised lysosomal enzyme function by lowering lysosomal pH (Henry et al., 2015). Furthermore, mutant LRRK2 astrocytes exhibited lysosomes with an enlarged morphology and reduced the capacity of lysosomes in the cell, however these deficits were rescued upon selective inhibition of LRRK2 kinase activity (Henry et al., 2015). In addition to showing localization with lysosomes, LRRK2 has also shown an association with endosomes and other vesicular bodies (Roosen and Cookson, 2016). Furthermore, iPSC-astrocytes harboring *LRRK2* or *GBA* mutations exhibited increased expression of α -synuclein caused a disruption in calcium signaling and altered metabolic activity (Sonninen et al., 2020). Whereas, primary PINK1-knock out mouse astrocytes exhibited mitochondrial dysfunction resulting in proliferation defects (Choi et al., 2013).

These studies are suggestive of a reactive astrocyte transformation in PD, whereby astrocytes not only suffer phagosomal-lysosomal defects, but contribute to disease pathogenesis through potential transfer of α-synuclein and pro-inflammatory processes. In addition, various PD-related mutations have a detrimental effect on homeostatic astrocyte functions within the CNS.

## 4 Conclusion

In summary, there is mounting evidence for the important role of astrocytes in disease pathogenesis of various neurodegenerative diseases. There is a critical loss of supportive astrocytic functions and in some contexts a toxic gain-of-function that cause astrocytes to not only affect the health of vulnerable neurons, but to develop a disease-specific astrocytic reactive phenotype. Overall, the presence of proteinopathies in astrocytes is significantly less well described and studied as compared to proteinopathies in a neuronal context, although there is evidence that in some diseases astrocytes not only develop disease-specific proteinopathies, but further propagate it (Lee et al., 2010; Fellner et al., 2013; Rannikko et al., 2015; Söllvander et al., 2016; Westergard et al., 2016; Smethurst et al., 2020; Wang and Ye, 2021). Therefore, it is unlikely that astrocytes are the direct cause of neurodegeneration, but rather active participants of disease. Moreover, these studies show that reactive transformations of astrocytes do have commonalities between diseases (e.g., pro-inflammatory activations, increased GFAP expression, trafficking and homeostasis disruptions). With more studies investigating transcriptional, biochemical, morphological, and metabolic variables, we are expected to find more defined disease and CNS region-specific astrocytic phenotypes. Thus, research on the role of astrocytes in specific disease contexts and regions of interest is essential to further understand the selective neurodegeneration and cell death leading to disease pathogenesis in these debilitating and fatal diseases.
