# Supplementary material for: The Fault in Our Astrocytes - cause or casualties of proteinopathies of ALS/FTD and other neurodegenerative diseases?
Source: Front Mol Med. 2023 Feb 16;3:1075805. doi: 10.3389/fmmed.2023.1075805 (PMC11334001; doi:10.3389/fmmed.2023.1075805)
Supplement: Supplementary file 1 [file Table1.DOCX]

Table 1. Neurodegenerative disease pathologies affecting astrocytes.

| Disease | Proteinopathy | Human astrocytic inclusions? | Affected astrocyte functions in human tissue and model systems | References |
| --- | --- | --- | --- | --- |
| ALS/FTD | **TDP-43** | Observed in the cytoplasm and radiating processes (identified as glial inclusions; unclear if astrocyte specific). | - Increase in GFAP expression - Transcriptional reactive phenotype - Impaired lipid metabolism, ß-adrenergic mediated aerobic glycolysis, and lactate production | Nishihira et al. (2008); Cooper-Knock et al. (2012); Peng et al. (2020); Velebit et al. (2020) |
|  | **FUS** | Cytoplasmic and nuclear inclusions (identified as glial inclusions; unclear if astrocyte specific). | - Increase in GFAP expression and secreted factors (TNFα) | Hewitt et al. (2010); Suzuki et al. (2012); Kia et al. (2018) |
|  | **SOD1** | Nuclear accumulations in the ventral horn of the spinal cord. | - Increase in GFAP expression - Reactive transformation - Secretion of motor neuron specific toxic factor - Impaired glutamate uptake | Nagai et al. (2007); (Yamanaka et al., 2008a; Yamanaka et al., 2008b); Forsberg et al. (2011); Guttenplan et al. (2020) |
|  | ***C9orf72* DPRs** | Detected in iPSC-derived astrocytes. | - Increase in GFAP expression - Reactive transformation - Impaired glutamate uptake - Impaired EV secretion - Oxidative stress | Shi et al. (2018); Birger et al. (2019); Varcianna et al. (2019); Guttenplan et al. (2020); Zhao et al. (2020); (Taha et al., 2022) |
|  | **TAU** | Observed in tufted astrocytes and astrocyte plaques. | - Increase in GFAP expression - Astrogliosis - reactive transformation - Morphological changes (hypertrophy) | Bang et al. (2015); Hallmann et al. (2017) |
| Alzheimer’s disease | **Amyloid-ß** | Astrocytes found surrounded around Aß plaques. | - Aß_42_ oligomers caused endosomal/lysosomal defects - Increase in GFAP expression | Pike et al. (1995); Bates et al. (2002); Sollvander et al. (2016) |
|  | **Neurofibrillary Tangles - Tau** | Observed in hilar astrocytes in the dentate gyrus. | - Impaired mitochondrial motility and function - Impaired trafficking network | Yoshiyama et al. (2003); Forman et al. (2005); Richetin et al. (2020) |
|  | **TDP-43** | Not found, glial cytoplasmic inclusions are found in transferrin-positive oligodendrocytes. | Not well described | Higashi et al. (2007) |
| Huntington’s disease | **HTT Expansion Variant** | HTT expansion variant fibrils found in the nucleus and cytoplasm of striatal astrocytes. | - Reactive transformation - Impaired glutamate uptake and K^+^ buffering - Morphological changes and hypertrophy - Electrophysiological defects, (depolarized membrane potentials and lower membrane conductance) - Altered functions in Ca^2+^ signaling, GPCR, and neurotransmitter regulation | DiFiglia et al. (1997); Cooper et al. (1998); Shin et al. (2005); Faideau et al. (2010); Tong et al. (2014); Jansen et al. (2017); Diaz-Castro et al. (2019) |
| Parkinson’s disease | **α-synuclein** | Observed within the substantia nigra, midbrain, amygdala, thalamus, striatum and cerebral cortex. | - Reactive transformation with pro-inflammatory response - Antigen-presenting phenotype (dependent on α-synuclein species) - Impaired phagosomal-lysosomal machinery - Impaired glutamate uptake | Wakabayashi et al. (2000); Braak et al. (2007); Lee et al. (2010); Rostami et al. (2017); Russ et al. (2021) |
